# Endogenous Retrotransposition Activates Oncogenic Pathways in Hepatocellular Carcinoma

**DOI:** 10.1016/j.cell.2013.02.032

**Published:** 2013-03-28

**Authors:** Ruchi Shukla, Kyle R. Upton, Martin Muñoz-Lopez, Daniel J. Gerhardt, Malcolm E. Fisher, Thu Nguyen, Paul M. Brennan, J. Kenneth Baillie, Agnese Collino, Serena Ghisletti, Shruti Sinha, Fabio Iannelli, Enrico Radaelli, Alexandre Dos Santos, Delphine Rapoud, Catherine Guettier, Didier Samuel, Gioacchino Natoli, Piero Carninci, Francesca D. Ciccarelli, Jose Luis Garcia-Perez, Jamila Faivre, Geoffrey J. Faulkner

**Affiliations:** 1 Division of Genetics and Genomics, The Roslin Institute and Royal (Dick) School of Veterinary Studies, University of Edinburgh, Easter Bush EH25 9RG, UK; 2 Cancer Biology Program, Mater Medical Research Institute, South Brisbane QLD 4101, Australia; 3 Department of Human DNA Variability, Pfizer-University of Granada and Andalusian Government Center for Genomics and Oncology (GENYO), 18007 Granada, Spain; 4 Edinburgh Cancer Research Centre, The University of Edinburgh, Western General Hospital, Crewe Road South, Edinburgh EH4 2XR, UK; 5 Department of Experimental Oncology, European Institute of Oncology (IEO), Via Adamello 16, 20139 Milan, Italy; 6 DIVET, School of Veterinary Medicine, University of Milan, Via Celoria, 20133 Milan, Italy; 7 INSERM U785, Centre Hépatobiliaire, Villejuif 94800, France; 8 Université Paris-Sud, Faculté de Médecine, Villejuif 94800, France; 9 RIKEN Yokohama Institute, Omics Science Center, 1-7-22 Suehiro-chô, Tsurumi-ku, Yokohama, Kanagawa 230-0045, Japan; 10 School of Biomedical Sciences, University of Queensland, Brisbane QLD 4072, Australia

## Abstract

LINE-1 (L1) retrotransposons are mobile genetic elements comprising ∼17% of the human genome. New L1 insertions can profoundly alter gene function and cause disease, though their significance in cancer remains unclear. Here, we applied enhanced retrotransposon capture sequencing (RC-seq) to 19 hepatocellular carcinoma (HCC) genomes and elucidated two archetypal L1-mediated mechanisms enabling tumorigenesis. In the first example, 4/19 (21.1%) donors presented germline retrotransposition events in the tumor suppressor *m*utated in *c*olorectal *c*ancers (*MCC*). *MCC* expression was ablated in each case, enabling oncogenic β-catenin/Wnt signaling. In the second example, *s*uppression of *t*umorigenicity *18* (*ST18*) was activated by a tumor-specific L1 insertion. Experimental assays confirmed that the L1 interrupted a negative feedback loop by blocking ST18 repression of its enhancer. *ST18* was also frequently amplified in HCC nodules from *Mdr2*^−/−^ mice, supporting its assignment as a candidate liver oncogene. These proof-of-principle results substantiate L1-mediated retrotransposition as an important etiological factor in HCC.

## Graphical abstract

**Graphical Abstract:**

**Figure:**
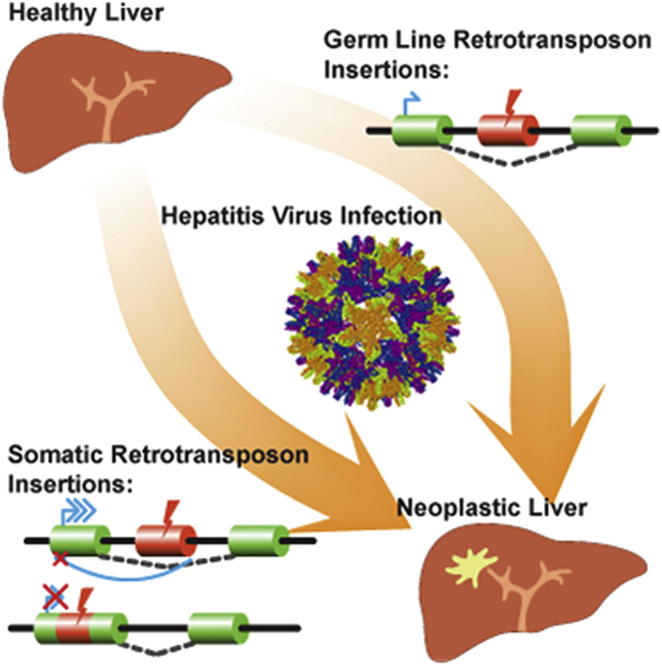

**Highlights:**

► L1 retrotransposons promote tumorigenesis in hepatocellular carcinoma (HCC) ► Germline L1 and *Alu* insertions in *MCC* activate β-catenin/Wnt signaling ► L1 mobilization in tumor cells accelerates transformation of the HCC genome ► A tumor-specific L1 insertion interrupts a negative feedback loop regulating *ST18*

## Teaser

L1 retrotransposons, which are widespread in the human genome, can mobilize and activate oncogenes in the livers of individuals infected with the hepatitis B or hepatitis C virus, promoting the development and growth of hepatocellular carcinoma. Genes identified by the L1 insertions present new options for cancer screening and intervention.

## Introduction

Liver cancer accounts for 9% of all cancer deaths worldwide and 12% in developing countries (Jemal et al., 2011). Pathological inspection indicates hepatocellular carcinoma (HCC) in ∼80% of liver tumors, with infection by hepatitis B virus (HBV) and hepatitis C virus (HCV) being the most prevalent risk factors, followed by chronic alcoholism (Jemal et al., 2011; Perz et al., 2006; Tateishi and Omata, 2012). Although early detection and monitoring of patients with liver cirrhosis can substantially improve 5 year survival rates, progression to advanced HCC reduces average life expectancy to less than 8 months (Llovet et al., 2008). As for other cancers, genome and exome resequencing have elucidated molecular pathways frequently perturbed in HCC (Guichard et al., 2012; Tateishi and Omata, 2012; Totoki et al., 2011), potentially enabling therapeutic intervention informed by the mutational signature of a given tumor. The capacity to catalog the full spectrum of genetic aberrations occurring in HCC is therefore of critical importance.

LINE-1 (L1) retrotransposons are a major source of endogenous mutagenesis in humans (Burns and Boeke, 2012; Levin and Moran, 2011). These mobile genetic elements utilize a “copy-and-paste” mechanism to retrotranspose to new genomic loci, with such success in germ cells that 500,000 L1 copies comprise ∼17% of the genome (Lander et al., 2001). Of these copies, only 80–100 are transposition competent, with distinct subsets of frequently active—or “hot”—L1s driving insertional mutagenesis in each individual genome (Beck et al., 2010; Brouha et al., 2003). Retrotransposon insertions can profoundly alter gene structure and expression (Cordaux and Batzer, 2009; Faulkner et al., 2009; Han et al., 2004; Levin and Moran, 2011) and have been found in nearly 100 cases of disease (Faulkner, 2011; Hancks and Kazazian, 2012). L1 activity is consequently suppressed in most somatic cells by methylation of a CpG island in the internal L1 promoter (Coufal et al., 2009; Swergold, 1990). By contrast, L1 is often hypomethylated in tumor cells, removing a key obstacle to retrotransposition (Levin and Moran, 2011).

Despite this failure to repress L1 transcription, only a handful of L1 insertions had been found in human tumors until very recently (Liu et al., 1997; Miki et al., 1992). High-throughput L1 integration site sequencing has since revealed 9 and 69 de novo L1 insertions, respectively, in lung and colorectal tumors (Iskow et al., 2010; Solyom et al., 2012), whereas cancer genome resequencing elucidated a further 183 tumor-specific L1 insertions in colorectal, ovarian, and prostate cancer (Lee et al., 2012). In this latter study, more than half of all insertions were found in a single colorectal tumor; the other individuals presented fewer than five tumor-specific L1 insertions on average. These data suggest L1 mobilization may be common in epithelial tumors, though the reasons for possible cell-of-origin restriction are currently unknown.

Tumor-specific L1 retrotransposition has not previously been observed in HCC. For several reasons it is, however, a logical cancer in which to expect L1 mobilization. First, HCC is epithelial in origin. Second, HBV and HCV infection are common in HCC; viruses can suppress host defense factors, such as APOBEC proteins, that control retrotransposon activation. APOBEC3G has been shown, for instance, to inhibit both HBV replication and endogenous retrotransposition (Esnault et al., 2005; Turelli et al., 2004). Third, liver inflammation precedes HCC and may, via cellular stress, stimulate retrotransposition (Fornace and Mitchell, 1986). Given these facts, we aimed to map L1 integration sites in HCC using retrotransposon capture sequencing (RC-seq) and assess their impact upon oncogenic and tumor suppressor pathways.

## Results

### Enhanced Retrotransposon Capture Sequencing

**Figure 1:**
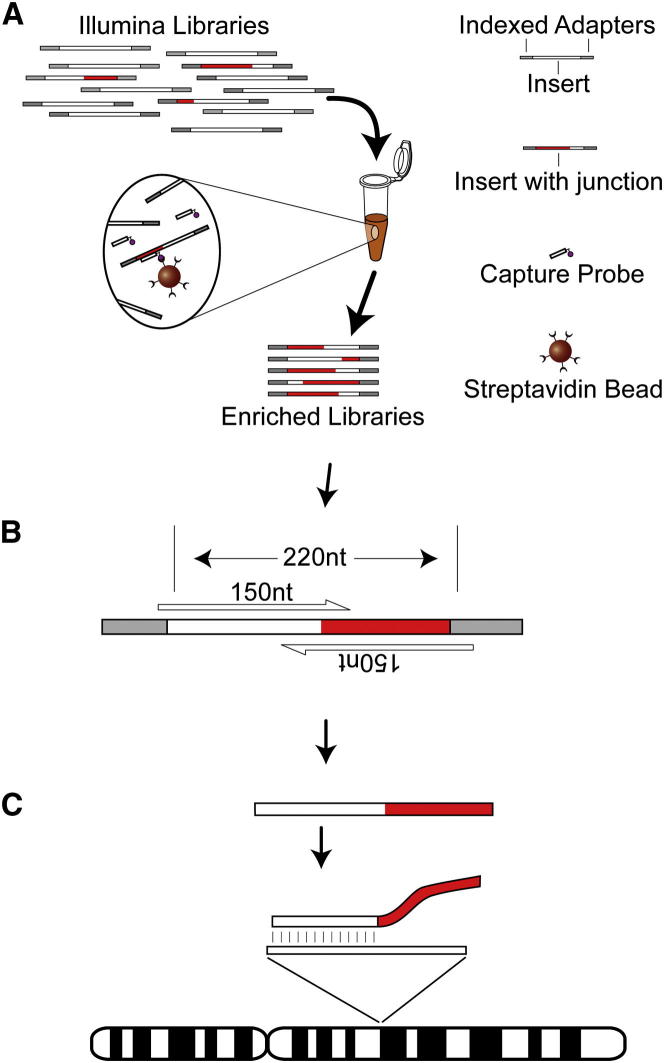
Enhanced RC-Seq (A) Multiplexed Illumina libraries are hybridized to liquid-phase sequence capture probes targeting the 5′ and 3′ ends of recently active human retrotransposons (Table S1). (B) Paired-end 150-mer sequencing of ∼220 nt inserts enables “contig” assembly of each read pair into a single read. (C) Assembled reads with a 5′ or 3′ section of an active retrotransposon at one end (highlighted in red) are retained. The opposite end is then aligned to the reference genome, indicating the position of known and novel insertions.

To test the hypothesis that L1 mobilizes in HCC, we applied an updated RC-seq protocol to 19 HCC tumors and matched adjacent liver tissue that were confirmed positive for HBV or HCV infection (Table 1). An earlier RC-seq design (Baillie et al., 2011) was modified to incorporate multiplex liquid-phase sequence capture (Figure 1A) using a refined probe pool (Table S1 available online) and a reduced insert size of ∼220 nt, which enabled high-confidence assembly of overlapping paired-end 150 nt reads (Figure 1B). This change simplified genomic alignment and, more importantly, enabled single-nucleotide resolution of retrotransposon integration sites (Figure 1C).

**Table 1:** Nonreference Genome Insertions Detected by RC-Seq

Donor	Gender	Virus	Age	Germline Insertions	Private Germline Insertions	Validated Tumor-Specific Insertions
12	M	HCV	65	2,082	202	3
15	M	HBV	53	1,845	216	1
21	M	HCV	51	2,019	271	0
29	M	HCV	52	1,602	44	0
32	M	HBV	73	1,681	100	0
33	F	HCV	57	1,982	234	2
35	F	HCV	78	1,786	96	0
42	F	HCV	67	1,594	43	0
47	M	HBV	61	1,581	77	2
48	M	HBV	35	1,744	212	0
49	M	HCV	68	1,644	58	0
60	M	HCV	48	1,570	33	0
62	M	HBV	33	1,750	153	0
70	M	HCV	55	1,673	82	0
86	F	HBV	56	1,701	50	0
89	M	HBV	60	1,739	163	4
95	M	HBV	54	1,773	88	0
106	M	HBV	60	2,141	48	0
116	M	HBV	62	1,532	71	0

F, female; M, male. Please see Tables S2 and S3 for supporting data and details.

After stringent filtering and mapping, an average of ∼2 million reads were retained per library with >95% identity to active L1, *Alu*, and SVA families, as well as the most recently active human LTR endogenous retroviruses (Table S2). Optimized sequence capture led to a 4-fold increase in reads aligned to nonreference genome L1s per library compared to previous RC-seq based on solid-phase arrays and similar sequencing depth (Baillie et al., 2011). The improved resolution of RC-seq also allowed us to discriminate a required minimum of two unique amplicons in support of any nonreference genome insertion (see Extended Experimental Procedures).

### Frequent Retrotransposition in the Human Germline

A total of 7,689 nonreference genome insertions were detected in 19 tumor (T) samples and 19 matched nontumor (NT) liver samples. Of these, we annotated 7,644 as putatively germline (Table S3) because of their presence in (1) databases of retrotransposon-induced polymorphisms (Beck et al., 2010; Ewing and Kazazian, 2010; Iskow et al., 2010; Wang et al., 2006), (2) pre-existing insertions annotated by pooled blood RC-seq (Baillie et al., 2011), (3) multiple individuals, or (4) nontumor liver. L1, *Alu*, SVA, and LTR-flanked retrotransposons comprised 13.5%, 81.8%, 4.3%, and 0.4% of germline insertions, respectively. As expected, L1-Ta and L1-pre-Ta (99.3%) and AluY (99.7%) were the main L1 and *Alu* subfamilies active in germ cells (Mills et al., 2007).

**Figure 2:**
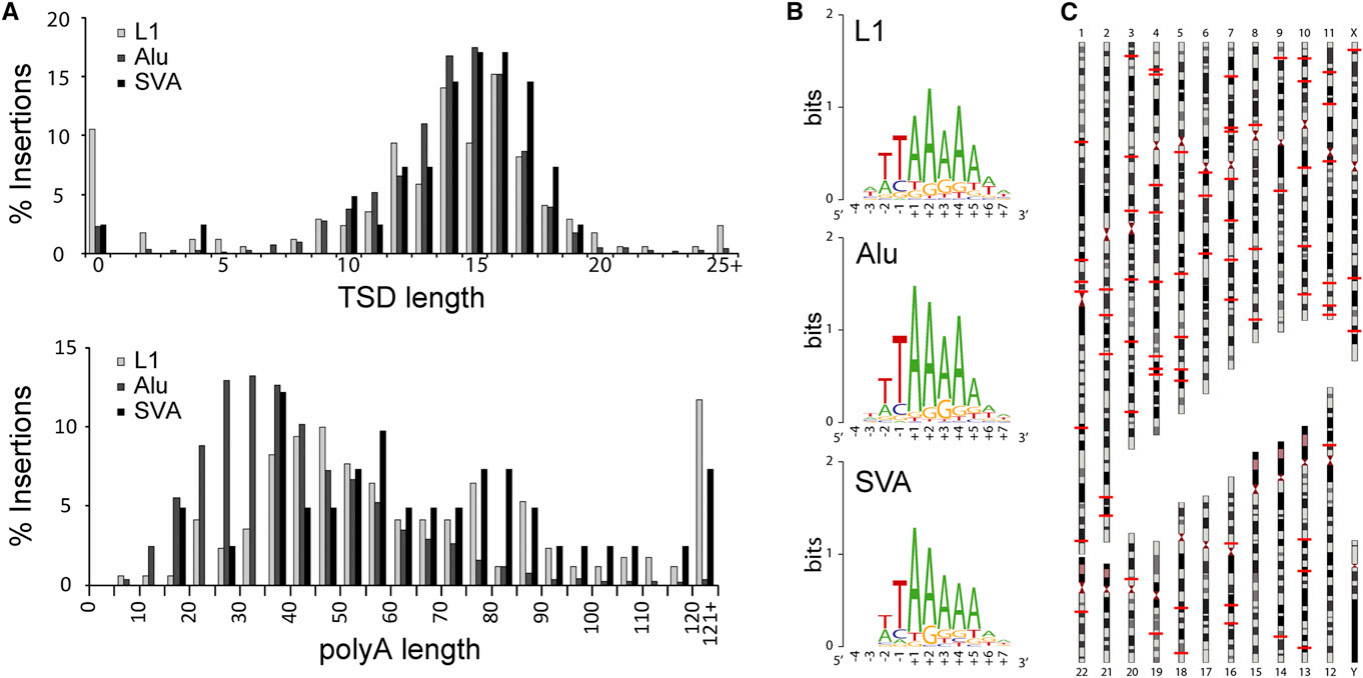
Characteristics of Recent Germline Retrotransposition in Humans (A) Distributions of target site duplication length and poly-A tail length for L1, *Alu*, and SVA. (B) Consensus sequence motifs (Crooks et al., 2004) at integration sites closely resembled the canonical L1 endonuclease recognition sequence. (C) Genomic positions (indicated by red lines) of 115 previously unobserved full-length L1 insertions detected at both termini by RC-seq. Please see Tables S4 and S5 for further supporting data.

A total of 2,241 germline insertions were found in only one individual each (Table 1 and Table S3) and were not annotated by the aforementioned retrotransposon polymorphism databases, suggesting that these were private or rare mutations or, alternatively, had occurred in early development (Garcia-Perez et al., 2007; Kano et al., 2009). RC-seq detected 1,489 (66.4%) insertions at both their 5′ and 3′ ends, enabling us to model the characteristic sequence features of L1-mediated retrotransposition. Without any additional sequencing, we were able to analyze insertions for the presence of target site duplications (TSDs), an L1-endonuclease recognition motif (Jurka, 1997), and a polyA tail (Figures 2A and 2B). These features consistently resembled target-primed reverse transcription (TPRT) for L1, *Alu*, and SVA, again illustrating the primary retrotransposition mechanism in germ cells (Cost et al., 2002; Jurka, 1997).

We also identified 160 previously undetected full-length (>99.9%) L1 copies, including 115 with paired 5′/3′ detection (Figure 2C; Table S4) and 82 each found in a single donor only. All were annotated as L1-Ta or pre-Ta. These potentially “hot” L1s added to a recent cohort of full-length L1 insertions found in six geographically diverse individuals via fosmid screening and sequencing (Beck et al., 2010). Of 68 L1 insertions reported by Beck et al. (2010), we detected 49 (72.1%), including 15/18 (83.3%) with an allelic frequency >5%. Of the 49 insertions common to both studies, 46 (93.9%) were base-pair identical in genomic position. These results confirm strong agreement between RC-seq and the conservative fosmid-based approach of Beck et al. (2010).

Each individual genome contained on average 244 nonreference genome L1 insertions, a figure 60% and 80% higher, respectively, than recent L1 insertion site sequencing on cell lines (Ewing and Kazazian, 2010) and single cells (Evrony et al., 2012). Therefore, to assess the RC-seq false-positive rate, we randomly selected 200 germline insertions (173 *Alu*, 14 L1, 11 SVA, and 2 LTR) for site-specific PCR validation (Table S5). Of these, we confirmed 197 (98.5%). The remaining three insertions (2 SVA and 1 *Alu*) occurred in repetitive genomic regions and were detected by multiple unique reads in at least ten different samples each, indicating that these may have represented PCR false negatives. These comparisons and experiments together demonstrate the sensitive and accurate mapping of bona fide retrotransposition events by RC-seq and further highlight ongoing L1 retrotransposition in the global human population (Beck et al., 2010; Ewing and Kazazian, 2010; Huang et al., 2010; Iskow et al., 2010).

### Activation of β-Catenin/Wnt Signaling via L1-Mediated Ablation of *MCC*

To assess the potential tumorigenic consequences of the identified nonreference genome insertions, we selected and validated, by insertion site PCR, 31 L1, *Alu*, and SVA insertions in genes generally implicated to play a causal role in cancer (Futreal et al., 2004) or specifically in HCC (Guichard et al., 2012), including L1 insertions in the proto-oncogene *ALK* and the tumor suppressor *FHIT* (Table S5). Quantitative RT-PCR indicated, however, that 28/31 of these germline insertions did not significantly perturb host gene expression in tumor or nontumor liver versus control liver from five unaffected individuals (data not shown).

Strikingly, the three remaining insertions all coincided with strong inhibition of the tumor suppressor *m*utated in *c*olorectal *c*ancers (*MCC*) (Higgins et al., 2007). *MCC* is expressed in liver (Senda et al., 1999) and regulates the oncogenic β-catenin/Wnt signaling pathway frequently activated in HCC (Fukuyama et al., 2008; Guichard et al., 2012; Totoki et al., 2011). In vitro experiments have established that siRNA knockdown of *MCC* mRNA dramatically increases β-catenin (CTNNB1) expression, whereas *MCC* overexpression inhibits cellular proliferation (Fukuyama et al., 2008; Matsumine et al., 1996). *MCC* is also an intriguing HCC candidate gene because of its genomic proximity to *APC*, a major tumor suppressor mutated in familial adenomatous polyposis preceding colorectal cancer (Groden et al., 1991; Kinzler et al., 1991). It is important to note that mutated *APC* occurs in <2% of HCC cases versus >60% of colorectal carcinomas (Guichard et al., 2012; Powell et al., 1992). We therefore hypothesized that germline retrotransposition events specifically inhibited *MCC* tumor suppressor function in liver. To test this prediction, we assessed the impact of each *MCC* mutation upon *MCC*, *APC*, and *CTNNB1* expression.

**Figure 3:**
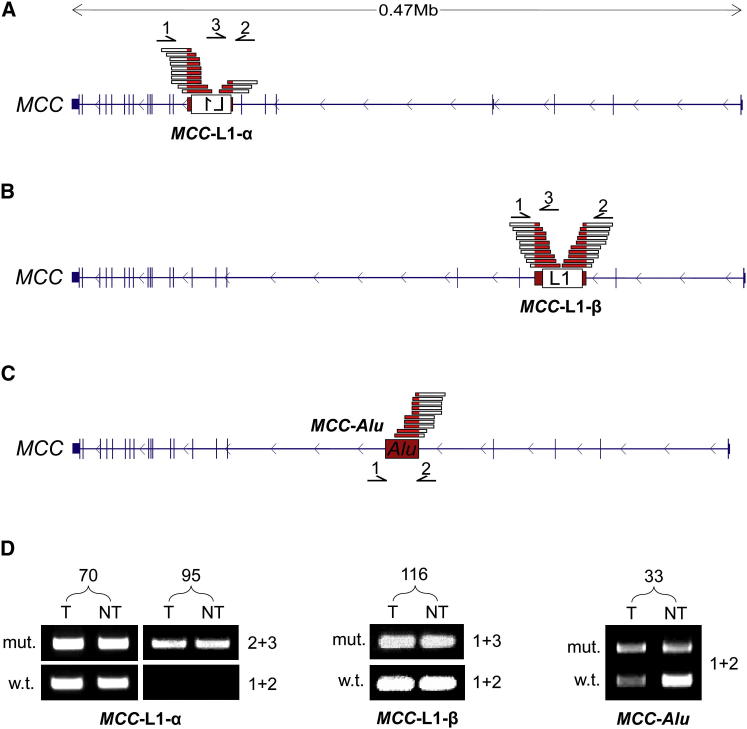
Structure and Validation of Germline L1 and *Alu* Insertions in *MCC* (A) *MCC* mutant allele *MCC*-L1-α: a 5.3 kb L1-Ta detected by RC-seq at its 5′ and 3′ ends in 70T, 70NT, 95T, and 95NT. The L1 was flanked by a 13 nt TSD. Primers used for PCR validation (1,2,3) and RC-seq reads (red/white bars) are indicated above the gene structure. Note: L1 not drawn to scale. (B) *MCC* mutant allele *MCC*-L1-β: a full-length (6 kb) L1-Ta detected by RC-seq at its 5′ and 3′ ends in 116T and 116NT. The L1 was antisense to *MCC* and was flanked by a 14 nt TSD. Primers are indicated as for (A). (C) *MCC* mutant allele *MCC*-*Alu*: an AluY detected by RC-seq at its 3′ end in 33T and 33NT. The AluY was antisense to *MCC*, had a 15 nt TSD, and bisected an annotated enhancer (Thurman et al., 2012). Primers (1,2) are indicated below the gene structure. (D) Insertion-site PCR validation confirmed that *MCC*-L1-α, *MCC*-L1-β, and *MCC*-*Alu* were present in the corresponding tumor and nontumor samples (Table S5). The wild-type allele was absent for donor 95, indicating a homozygous L1 insertion.

Three germline retrotransposon insertions were found in *MCC*. The first of these, labeled *MCC*-L1-α, comprised a 5.3 kb L1-Ta oriented in sense to *MCC* in donors 70 and 95 (Figure 3A). Another L1-Ta, labeled *MCC*-L1-β, was full-length (6 kb), occurred at a different genomic position in donor 116, and was oriented antisense to *MCC* (Figure 3B). Finally, in donor 33, we found an AluY (*MCC*-*Alu*; Figure 3C) inserted in an ENCODE-delineated enhancer (Thurman et al., 2012). Insertion site PCR revealed that *MCC*-L1-α was heterozygous in donor 70 and homozygous (or possibly hemizygous) in donor 95, whereas *MCC*-L1-β and *MCC*-*Alu* were heterozygous in donor 116 and donor 33, respectively (Figure 3D).

**Figure 4:**
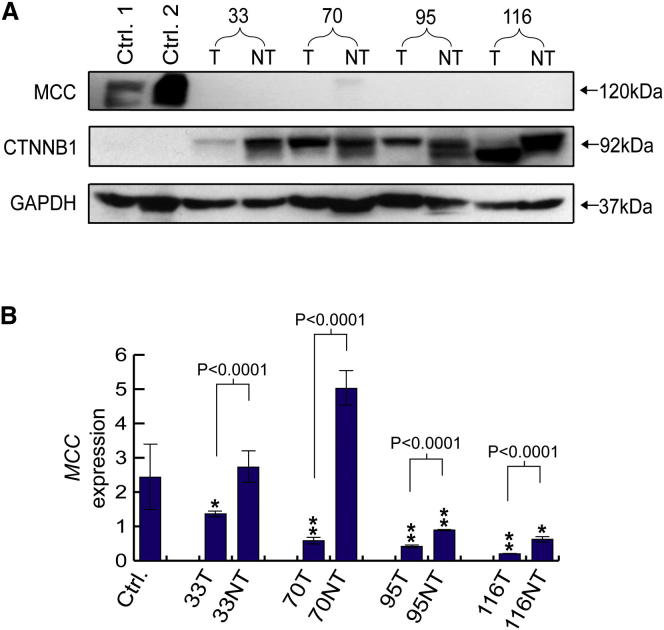
Downregulation of *MCC* (A) Relative expression of MCC and CTNNB1 in control liver tissue compared to tumor and nontumor liver tissue from donors 33, 70, 95, and 116. An immunoblot performed against anti-MCC, anti-CTNNB1, and anti-GAPDH (loading control) antibodies detected strong MCC expression only in controls and strong CTNNB1 expression only in *MCC* mutant donors. MCC was also detected weakly in donor 70NT, in line with qRT-PCR results shown in (B). Expected protein molecular weights are marked on right. Note: anti-MCC and anti-CTNNB1 antibodies produce double bands. See Figure S1 for donor 116 CTNNB1 immunohistochemistry. (B) Downregulation of *MCC* transcription in *MCC* mutant donors: qRT-PCR revealed that *MCC* mRNA was significantly reduced compared to control liver tissue in donors 33 (tumor only), 70 (tumor only), 95 (tumor and nontumor), and 116 (tumor and nontumor). ^∗∗^p < 0.002 and ^∗^p < 0.02, two-tailed t test, df = 19. In all four donors, MCC was also strongly downregulated in tumor versus nontumor samples (p < 0.0001, two-tailed t test, df = 10). Data are presented as mean ± SD. See Figure S2 for *APC* qRT-PCR.

**Figure S1:**
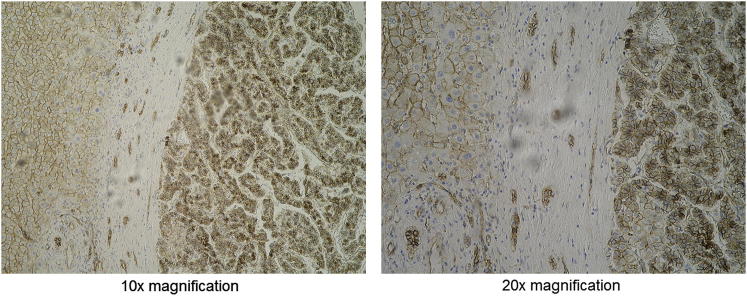
Immunohistochemistry Reveals Aberrant CTNNB1 Expression in Donor 116, Related to Figure 4 Sections taken from the nontumor/tumor interface indicated CTNNB1 expression in the plasma membrane and cytoplasm of many non-tumor cells and strong CTNBB1 expression in the plasma membrane, cytoplasm and nuclei of most tumor cells. Non-tumor and tumor are arranged left-to-right in each interface image. Images were taken at 10x (left) and 20x (right) magnification. Brown indicates CTNNB1, blue indicates nuclei.

An immunoblot indicated that MCC was dramatically less abundant in tumor and nontumor samples from all four donors compared with control liver tissue (Figure 4A). By contrast, CTNNB1 was expressed much more strongly in the affected donors than in controls (Figure 4A). This inverse relationship was consistent with MCC suppression of CTNNB1 through protein-protein interactions, as reported elsewhere (Fukuyama et al., 2008). As a corroborating example, immunohistochemistry performed on tumor and nontumor tissue from donor 116 confirmed cytoplasmic CTNNB1 accumulation (Figure S1), a strong indicator that the factors controlling CTNNB1 expression outside of the plasma membrane were absent and that many cells had entered a proliferative state (Nhieu et al., 1999).

Quantitative RT-PCR indicated that *MCC* transcription was severely reduced (p < 0.02–p < 0.002, t test, degrees of freedom [df] = 19) in all four tumors compared to normal liver (Figures 4B). *MCC*-L1-α and *MCC*-L1-β strongly suppressed *MCC* expression in donor 95 and donor 116’s nontumor liver, respectively (Figure 4B). *MCC* was also significantly downregulated in tumor versus nontumor in all four individuals (p < 0.0001, t test, df = 4). Capillary sequencing of each *MCC* exon revealed only one missense mutation, a homozygous SNP (570 A > G) in donor 33 MCC exon 5, producing an Arg > Lys substitution in the putative CTNNB1 binding domain of MCC (Fukuyama et al., 2008). Therefore, *MCC*-L1-α, *MCC*-L1-β, and *MCC*-*Alu* were the primary enactors of *MCC* transcriptional inhibition, potentially assisted by other modifications to *MCC* or its upstream regulatory pathway.

**Figure S2:**
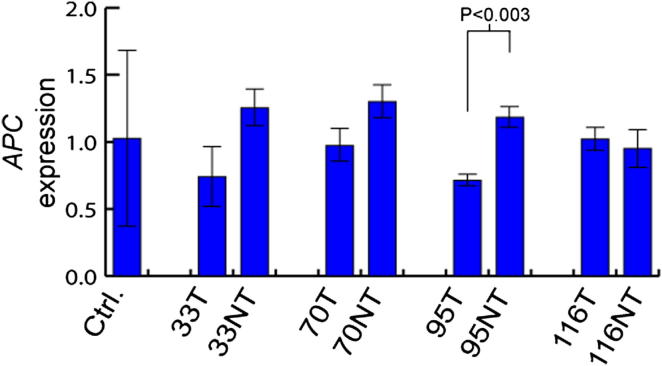
*APC* Expression Was Not Affected by Nearby *MCC* Mutations, Related to Figure 4 qRT-PCR revealed no significant changes in *APC* transcription in tumor or non-tumor tissue versus normal control liver (two-tailed t test, df = 16). Changes in *APC* expression in tumor versus non-tumor were far less pronounced than for *MCC* (Figure 4B), with only one individual (donor 95) showing a significant reduction in expression (p < 0.003, two-tailed t test, df = 4). Data are mean +/− SD.

Finally, we performed qRT-PCR to evaluate *APC* transcription coincident with mutated *MCC*. We found no significant differential transcription of *APC* in tumor or nontumor liver from the four affected donors versus normal liver controls (Figure S2). In donor 95, *APC* was downregulated significantly in tumor versus nontumor (p < 0.003, t test, df = 4) but only by 30% versus normal liver controls. By contrast, *MCC*-L1-α, the homozygous L1 insertion in donor 95, severely reduced *MCC* transcription in both tumor (−83%) and nontumor (−63%) samples compared with normal liver controls (Figure 4B), demonstrating that the primary effect of *MCC*-L1-α was on *MCC* rather than *APC*. These data in sum confirmed that (1) L1-mediated retrotransposition in *MCC* specifically repressed *MCC* and not *APC* and (2) *CTNNB1* was strongly induced in all four affected individuals, indicating activation of a major HCC oncogenic pathway.

### Somatic L1 Mobilization in HCC

Forty-five nonreference genome insertions were annotated as tumor specific. These consisted of 17 L1, 27 *Alu*, and 1 SVA. We first validated each L1 insertion with insertion site PCR, including capillary sequencing of their 5′ and 3′ ends (Table S6). All 17 L1s successfully amplified; 12 confirmed as tumor-specific, and 5 were found in both tumor and nontumor liver. Further examination of the tumor-specific set revealed uniform usage of the degenerate L1 endonuclease motif highlighted in Figure 2B. In two examples, PCR amplification of the 5′ junction was repeatedly unsuccessful, preventing TSD characterization, an outcome possibly due to gross genomic abnormality at the L1 insertion site (Gilbert et al., 2002). Eight of the other integration sites incorporated TSDs, whereas the remaining two examples involved small genomic deletions 3′ of the insertion site and no TSD. Somatic L1 mobilization occurred in donors 12, 15, 33, 47, and 89 (Table 1), with the latter individual presenting four insertions. Two L1 copies (chr11:60136439 and chrX:99180431) were greater than 5.3 kb in length, but no insertions were full-length. All 12 somatic L1 insertions were from the L1-Ta subfamily.

We next evaluated 13 *Alu* insertions and the single SVA insertion found only in tumor, using insertion site PCR. In all cases, amplification occurred in both tumor and adjacent liver DNA, indicating germline insertions. Our primary explanation for this result is that there are several thousand potentially active AluY copies in the genome, compared to fewer than 100 active L1s (Bennett et al., 2008; Brouha et al., 2003). As seen previously, the RC-seq read count per *Alu* is consequently 75% lower than for L1 (Baillie et al., 2011), making false-negatives in the nontumor control more likely for *Alu* than for L1. A secondary explanation is that chromosomal gain is very common in HCC (Guichard et al., 2012), increasing the probability that some germline insertions are detected in tumor but not in adjacent nontumor liver. A final possibility is that mutations in individual precancerous cells are clonally amplified in tumors and are called as tumor-specific by RC-seq and germline by insertion site PCR. However, this was unlikely, as we consistently observed strong PCR amplification in both tumor and nontumor liver in these cases. Consequently, RC-seq reliably identifies new L1, *Alu*, and SVA mobilization events but requires insertion site PCR to annotate tumor-specific insertions.

**Figure S3:**
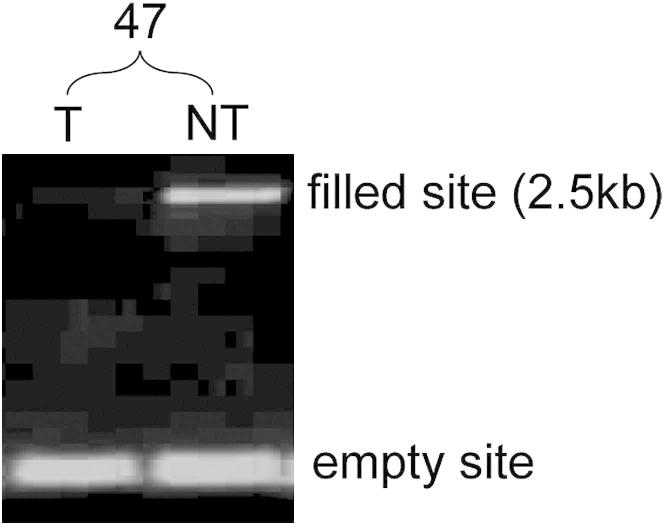
Detection of a Liver-Specific L1 Insertion, Related to Figure 5 The L1 (filled site) was located in an intergenic region (chr13:27423763) and was detected by site-specific PCR only in 47NT while the empty site was found in 47T and 47NT, indicating a heterozygous, liver-specific insertion. See Table S6 for further details.

In recent work, we reported somatic L1 mobilization in the normal brain but did not evaluate other organs (Baillie et al., 2011). For the current study, somatic L1 insertions in nontumor liver were considered difficult to evaluate because of the frequent occurrence of chromosomal loss in tumors. In this scenario, germline L1 insertions may be deleted in tumor but retained in nontumor liver and called somatic events. Nonetheless, we identified 21 L1 insertions restricted to nontumor liver in the set putatively annotated as germline and as a proof-of-principle experiment selected an example (chr13:27423763) for insertion site PCR and capillary sequencing (Table S6). This 2.5 kb L1-Ta insertion was detected only in liver and, interestingly, had a long (127 nt) TSD (Figure S3). A germline L1 insertion deleted in tumor cells would reasonably be expected to be detected in the nontransformed cells (e.g., lymphocytes) infiltrating a tumor (Unitt et al., 2005). Therefore, this very likely represented a bona fide liver-specific somatic L1 insertion in the preneoplastic liver of donor 47. Consequently, hepatocytes, or their progenitor cells, may support limited somatic L1 mobilization, though the contribution of this activity to malignancy remains unclear.

### L1 Hypomethylation Enables Tumor-Specific Mobilization

**Figure 5:**
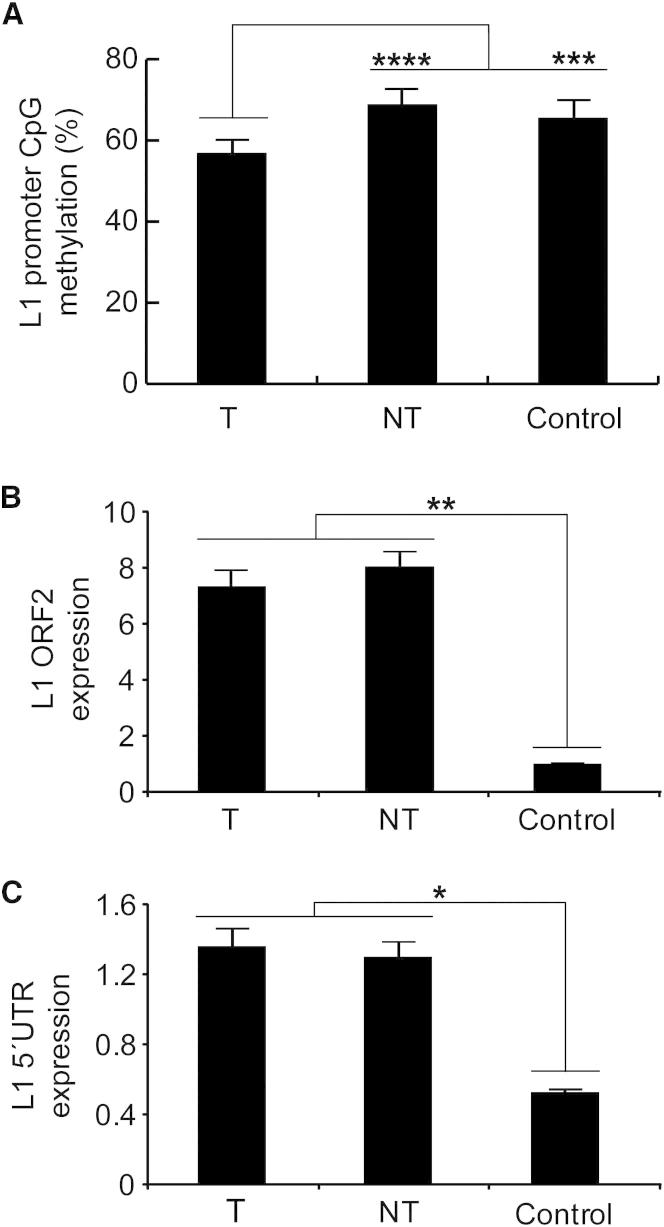
L1 Promoter Activation in HCC (A) Bisulphite analyses in HCC patients versus controls revealed a significant decrease in L1 promoter methylation in tumor samples. Each column represents the methylation of 20 CpG residues found within the internal L1-Ta promoter. Values are presented as the mean percent of CpG methylation ± SEM (^∗∗∗^p < 0.0005, ^∗∗∗∗^p < 2.5 × 10^−18^, chi-square test). Please see Figure S4 for detailed analysis. (B) TaqMan qRT-PCR measurement of L1 ORF2 indicated significantly increased L1 transcription in tumor and adjacent matched liver tissue versus controls. Data for each group (tumor, nontumor, and control) were pooled and presented as mean ± SEM (^∗∗^p < 0.003, two-tailed t test, df = 22, Bonferroni correction). (C) As for (B), except observed at the L1 5′ UTR (^∗^p < 0.006). Please see Figure S3 for an example of a somatic L1 insertion in nontumor liver.

**Figure S4:**
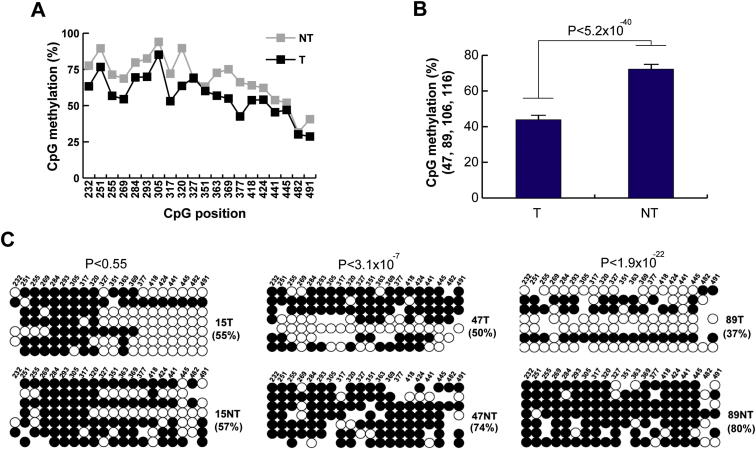
L1 Promoter Methylation in Tumor, Nontumor Liver, and Control Liver, Related to Figure 5 (A) Global L1 promoter methylation analysis in tumor (T) and nontumor (NT) bisulphite converted genomic DNAs. The graph shows percentage methylation for each of 20 distinct CpG dinucleotides for 8 grouped tumor samples and 8 grouped adjacent nontumor controls. Donors 15, 47, 48, 62, 89, 95, 106 and 116 were included. The *x* axis indicates the position of each CpG dinucleotide, using L1.3 as a reference (accession number L19088.1). (B) The L1 promoter is strongly hypomethylated in tumors from donors 47, 89, 106, and 116. Each column represents the overall percentage methylation for 20 CpG residues within the L1-Ta promoter. Data are mean +/− SEM. A strong difference was observed in tumor versus nontumor (p < 5.2x10^−40^, chi-square test). (C) L1 promoter methylation in HCC T/NT pairs. Shown are the 7 clones with the highest sequence similarity to L1.3. In the cartoon, white and black circles represent unmethylated and methylated CpG dinucleotides respectively. Mutated CpG dinucleotides are represented by the absence of a circle. P values were calculated using chi-square tests.

To assess whether L1 activity and L1 methylation state were correlated in HCC samples, we performed bisulphite conversion of gDNA and capillary sequenced the CpG island present in the canonical L1 promoter. Eight tumors (15T, 47T, 48T, 62T, 89T, 95T, 106T, and 116T) matched adjacent liver samples, and control liver samples were analyzed. In the tumor group, 54.8% of L1-promoter CpG dinucleotides were methylated, compared with 69.2% in nontumor liver, a strongly significant difference (p < 2.5 × 10^−18^, chi-square test, n = 8) (Figure 5A). On average, all but one CpG was hypomethylated in tumor, with the remaining CpG being equally methylated in tumor and nontumor liver (Figure S4A). Hypomethylation was not observed in grouped adjacent nontumor liver tissue versus controls.

As shown in Figure S4B, a subset of four individuals (donors 47, 89, 106, and 116) presented much stronger L1-promoter hypomethylation in their tumor (40.5%) versus nontumor liver (72.3%) samples compared with the remaining individuals (69.2% versus 66.1%). The three individuals with tumor-specific L1 insertions and L1 methylation data (donors 15, 47, and 89) yielded a strong correlation between L1 hypomethylation percentage and tumor-specific L1 insertion count (r = 0.97; n = 3). Donor 89 exhibited the strongest tumor-specific L1 hypomethylation and also had the most tumor-specific L1 insertions (Figure S4C). Donor 15 showed only tumor-specific hypomethylation distal to the L1 5′ end, whereas donors 47 and 89 were hypomethylated across the L1 promoter (Figure S4C).

Hypomethylation of the L1 promoter enables transcription of full-length L1 mRNAs that are translated to form the L1 mobilization machinery (Ostertag and Kazazian, 2001a). We therefore used cDNA synthesized with L1-specific primers (Wissing et al., 2012) to quantify L1 expression levels by TaqMan qRT-PCR. In this analysis, we measured L1 mRNA levels using primers targeting L1 ORF2 (Figure 5B) and the L1 5′ UTR (Figure 5C). In both cases, significant enrichment was observed in tumor and nontumor versus normal controls (p < 0.003 for ORF2, p < 0.006 for 5′ UTR, t tests, df = 22). Together, these data showed that L1 was activated and transcribed in HCC, coincident with hypomethylation of the L1 promoter.

### *ST18* Activated by a Tumor-Specific L1 Insertion

**Figure S5:**
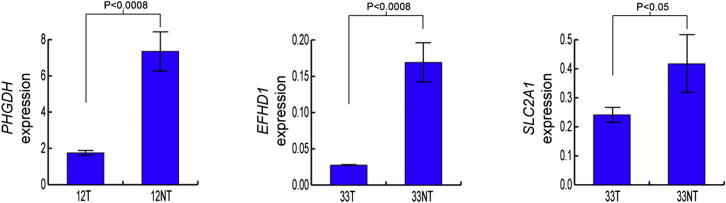
*PHGDH*, *EFHD1*, and *SLC2A1* Are Expressed in Liver and Downregulated by Intragenic, Tumor-Specific L1 Insertions, Related to Figure 6 qRT-PCR revealed significantly reduced transcription in tumor versus nontumor in all three genes. P values were calculated using a two-tailed t test (df = 4). Three other genes contained intronic, tumor-specific L1 insertions: *SLC5A8*, *STXBP5L* and *ST18* (Table S6). Of these, *SLC5A8* and *STXBP5L* were not expressed in liver according to our qRT-PCR results and *ST18* was upregulated in tumor (Figure 6D). Values were normalized to *TBP*. Data are mean +/− SD.

Tumor-specific L1 insertions were observed in six protein-coding genes (Table S6). Quantitative RT-PCR indicated that two of these genes (*STXBP5L* and *SLC5A8*) were not expressed in liver. The expression of three other genes was reduced 2-fold to 6-fold in tumor versus adjacent liver (p < 0.05, t test, df = 4), including a 3′ UTR insertion in *SLC2A1* and intronic insertions in *PHGDH* and *EFHD1* (Figure S5). These examples resemble those seen in other cancers in which intragenic L1 insertions in tumors coincided with reduced host gene expression (Lee et al., 2012). To our knowledge, downregulation of *SLC2A1*, *PHGDH*, or *EFHD1* has not previously been associated with cancer.

The remaining tumor-specific L1 insertion occurred in donor 47 and was associated with activation of the transcriptional repressor *s*uppression of *t*umorigenicity *18* (*ST18*), a member of the MYT1 zinc-finger transcription factor family (Yee and Yu, 1998). Contrasting reports depict *ST18* as a tumor suppressor and as an oncogene in different cancers (Jandrig et al., 2004; Steinbach et al., 2006). *ST18* is, however, very poorly expressed in liver (Jandrig et al., 2004), making it unlikely to act as a tumor suppressor in this context. Ectopic host gene expression was an unusual consequence of an L1 insertion given that these events are usually repressive (Han et al., 2004). As such, we hypothesized that *ST18* was a candidate liver oncogene activated via an unknown mechanism triggered by an intronic L1 insertion.

**Figure 6:**
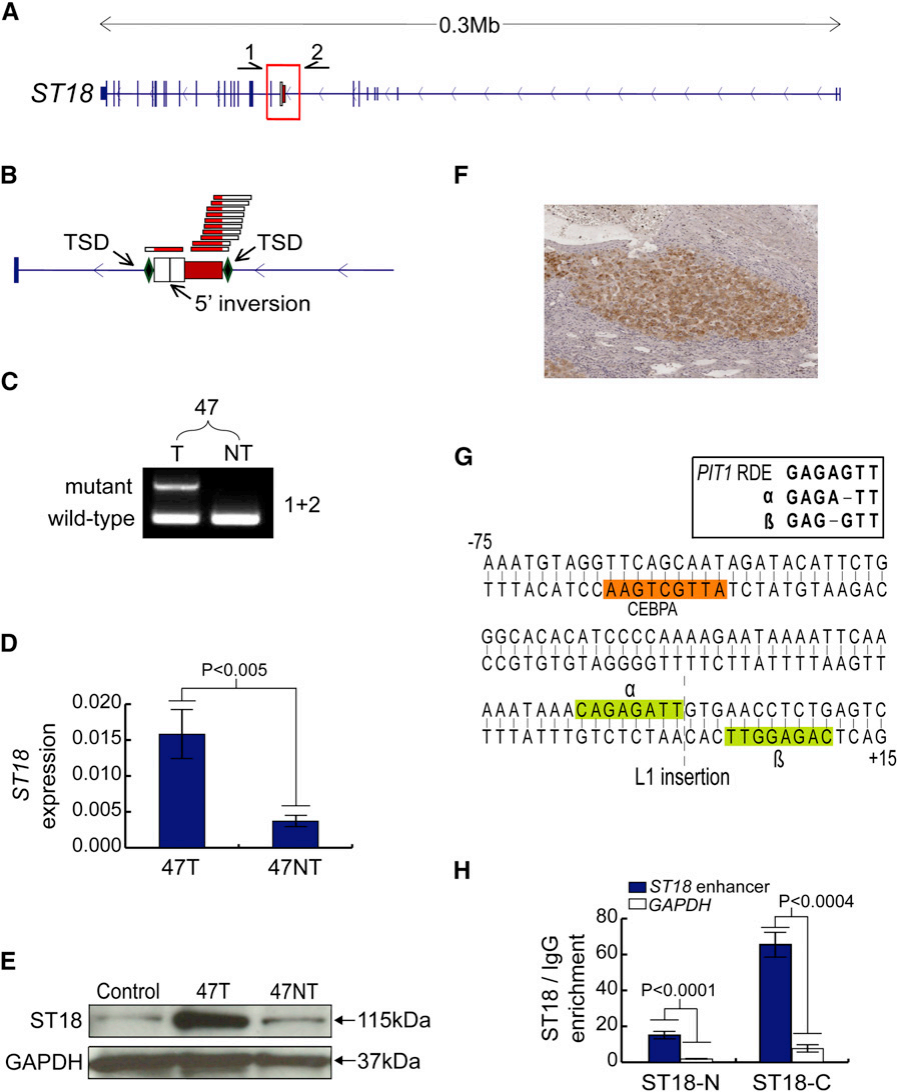
A Tumor-Specific L1 Insertion Causes Induction of *ST18* (A) *ST18* mutant allele: a 0.4 kb L1-Ta insertion antisense to *ST18*. Primers used for PCR validation (1,2) are indicated above the gene. (B) L1 insertion, magnified view: RC-seq detected the L1 5′ and 3′ termini, indicating a 17 nt TSD and a 5′ inversion. (C) Insertion-site PCR validation: the L1 was detected only in 47T, whereas the empty site was found in both 47T and 47NT. (D) qRT-PCR: *ST18* was upregulated 4-fold in 47T versus 47NT (^∗^p < 0.005, two-tailed t test, df = 4). Data are presented as mean ± SD. (E) ST18 immunoblot: ST18 (115 kDa) was enriched in 47T versus 47NT and normal liver controls. (F) ST18 immunohistochemistry: accumulation of ST18 (brown) was observed in tumor nodules compared to surrounding nontumor regions. Nuclei were stained with hematoxylin (blue). (G) A palindromic sequence motif was bisected by the L1. Each 8 nt unit (α and β, light green) contained a subsequence 1 nt different to a *PIT1*-enhancer motif known to bind MYT1 (Rhodes et al., 1993). A second motif −58 bp from the L1 integration site matched the consensus CEBPA binding motif (orange). (H) ChIP followed by quantitative real-time PCR in Huh7 cells confirmed enrichment for ST18 bound to the putative *ST18*-enhancer element illustrated in (G), compared to GAPDH. Data from antibodies targeting both the N termini and C termini of ST18 are shown. Significance values were calculated using two-tailed t tests (df = 4). Data are presented as mean ± SD. Please see Tables S6 and S7 and Figures S5 and S6 for further information regarding tumor-specific L1 insertions and additional *ST18* characterization.

Initial data from RC-seq indicated a heavily 5′ truncated, 410 bp L1-Ta arranged antisense to *ST18* (Figure 6A). The integration site incorporated a 17 nt TSD, a degenerate L1 endonuclease motif (GC/AAAA), and a 112 bp 5′ inversion of the L1 (Figure 6B), consistent with twin priming (Ostertag and Kazazian, 2001b). We then confirmed these features by PCR amplification and capillary sequencing of the L1 5′ and 3′ junctions, indicating a tumor-specific L1 insertion (Figure 6C). PCR on DNA extracted from three distinct biopsies taken from the same tumor detected the L1 in all three regions, suggesting clonal amplification of tumor cells with the L1 mutant *ST18*.

As noted above, qRT-PCR indicated that *ST18* expression was significantly increased in tumor versus adjacent nontumor liver (p < 0.005, t test, df = 4) (Figure 6D). To corroborate this result, we performed an immunoblot and immunohistochemistry with an anti-ST18 antibody and found ST18 was indeed ectopically expressed in donor 47 tumor (Figures 6E and 6F). Chromosomal gain and regional copy number variation (CNV) have previously been reported for chromosome 8q, the genomic region containing *ST18* (Guichard et al., 2012). However, quantitative real-time PCR on gDNA indicated no *ST18* CNV in donor 47 tumor. Thus, tumor cells containing the *ST18* L1 mutation were clonally amplified without CNV of the *ST18* locus, followed by *ST18* transcriptional activation.

In response, we predicted that *ST18* was activated by insertional mutagenesis of a *cis*-regulatory element proximal to the L1. In silico analysis of the L1 integration site indicated that it bisected a palindromic motif containing two 8 bp units differing by one nucleotide and separated by 3 bp (Figure 6G). The probability of a random insertion in this motif, even allowing for a mismatch in the palindrome and a generous gap of ≤11bp, was less than 1/1,000 (permutation test). Intriguingly, each unit was only one nucleotide different to a strong MYT1 binding motif found in the enhancer of *PIT1* (Rhodes et al., 1993). Previous experiments predicted that these units would bind MYT1 with reduced efficiency (Jiang et al., 1996), though transcription factors incorporating two zinc-finger domains, as for MYT1, are known to greatly gain efficiency through binding tandem DNA motifs (Yee and Yu, 1998). The putative MYT1 binding site was proximal to a strong binding site for CEBPA, a transcription factor enriched in liver and known to bind active enhancers (Johnson et al., 1987).

Based on this computational analysis, we predicted that the L1 bisected an enhancer normally bound to the zinc fingers of the ST18 MYT1 domain. To test this experimentally, we performed chromatin immunoprecipitation (ChIP) of DNA bound to the ST18 protein in Huh7 cells, followed by PCR amplification of the putative *ST18* enhancer. This assay confirmed that, absent an L1 insertion, ST18 was preferentially bound to its own enhancer (p < 0.0004, t test, df = 4) (Figure 6H). An L1 insertion in the ST18 binding site would reasonably be expected to displace this repressive mark from the enhancer. Thus, we experimentally validated a model of *ST18* activation in which a negative feedback loop was interrupted by a tumor-specific L1 insertion.

**Figure S6:**
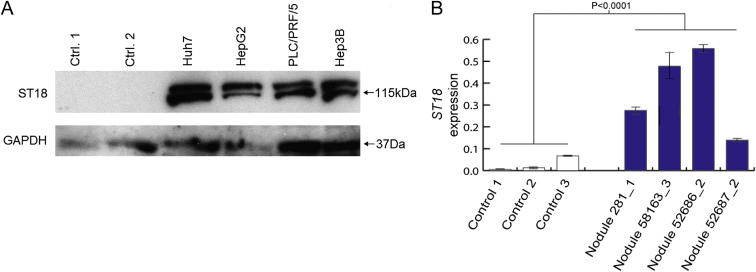
Pronounced *ST18* Expression in HCC Model Systems, Related to Figure 6 (A) An immunoblot for ST18 (115kDa) found enrichment in 4 analyzed human HCC cell lines compared to normal liver controls. (B) qRT-PCR indicated *ST18* transcription was strongly upregulated in four *Mdr2*^−/−^ mouse nodules with amplified *ST18* (Table S7) versus wild-type liver (p < 0.0001, two-tailed t test, df = 19). Values were normalized to *TBP*. Data are mean +/− SD.

Finally, in view of the clonal amplification of tumor cells containing ectopically expressed *ST18*, we engaged complementary in vitro and in vivo experimental models to assess *ST18* oncogenic function in HCC. Although *ST18* is poorly expressed in liver, we found it to be abundant in several liver cancer cell lines (Figure S6A). We then determined the frequency of *ST18* CNV in an *Mdr2*^−/−^ mouse model of inflammation-driven HCC. TaqMan quantitative real-time PCR detected *ST18* amplification in 4/23 *Mdr2*^−/−^ HCC nodules and no deletions (Table S7). A disproportionately high percentage of advanced tumors (75%) presented *ST18* amplification. *ST18* expression was also significantly higher in nodules with amplified *ST18* compared with wild-type mouse liver (p < 0.0001, t test, df = 19) (Figure S6B). These experiments demonstrate concordance of frequent *ST18* amplification and upregulation in human and mouse models of HCC, results consistent with *ST18* functioning as a candidate liver oncogene.

## Discussion

The present study highlights endogenous L1-mediated retrotransposition in the germline and somatic cells of HCC patients. We report two archetypal mechanisms revealing *MCC* and *ST18* as HCC candidate genes. *MCC* is, for the many reasons highlighted above, a highly plausible liver tumor suppressor. Four out of 19 individuals studied here, including two cases each of HBV and HCV infection, presented distinct germline L1 or *Alu* insertions contributing to *MCC* suppression in tumor and nontumor liver tissue. Strong upregulation of CTNNB1 in all four donors was consistent with prior observations that CTNNB1 is inhibited by MCC (Fukuyama et al., 2008). It is also interesting that *MCC*-L1-α was homozygous in donor 95, and therefore, *MCC* was almost certainly downregulated in the liver of this patient prior to HBV infection, i.e., preceding viral challenge, cirrhosis, and tumorigenesis.

We also demonstrate that *MCC* transcriptional repression in all four affected donors was exclusive of *APC*. Mutated *APC* is common in colorectal cancer but rare in HCC (Guichard et al., 2012; Powell et al., 1992). Even in colon, *MCC* presents numerous properties of a tumor suppressor (Bouwmeester et al., 2004; Fukuyama et al., 2008; Kohonen-Corish et al., 2007; Matsumine et al., 1996). Indeed, a Sleeping Beauty transposon mutagenesis screen using a mouse model of colorectal cancer found specific mutations in *MCC* and *APC* at a 1:9 ratio (Starr et al., 2009). Very recently, exome resequencing identified sporadic *MCC* point mutations in HCC (Guichard et al., 2012). Thus, *MCC* has potential to act as a liver tumor suppressor independent of *APC*, and our results support this potentially pivotal line of enquiry.

Tumorigenic retrotransposition in somatic cells was first observed 20 years ago, coincidentally in the *APC* gene of an individual with colorectal cancer (Miki et al., 1992). High-throughput sequencing has since provided the means to test whether tumor-specific retrotransposition is a common feature of cancer. Our results indicate that L1 mobilization occurs in a minority of HCC tumors, adding to the list of epithelial cancers (lung, colon, ovarian, and prostate) known to support the phenomenon (Iskow et al., 2010; Lee et al., 2012; Miki et al., 1992; Solyom et al., 2012). Although transformed tumor cells, including liver cancer cell lines, support frequent transgenic L1 mobilization (Moran et al., 1996), it is unknown whether endogenous L1 activation precedes neoplastic transformation in vivo. For this reason, it was interesting that L1 transcription was found in liver tissue adjacent to tumors, in addition to an example of somatic L1 mobilization. Finally, in a small cohort of tumor-specific L1 insertions, we identified mobilization via TPRT, twin priming, and a third mechanism resulting in a small deletion and no TSD, as reported elsewhere (Gilbert et al., 2002). These observations highlight the multiple routes by which L1 mobilization alters the tumor cell genome.

The results presented here corroborate recent data generated via whole-genome sequencing of other cancers. As in our study, Lee et al. (2012) described tumor-specific L1 insertions bearing the hallmark features of TPRT and also found intragenic L1 insertions in differentially expressed genes (Lee et al., 2012). One distinct feature of the current study is our discovery that germline L1 and *Alu* insertions significantly perturb expression of genes relevant to HCC. Another advance is our explanation for the occasional activation of host genes by tumor-specific L1 insertions, based on an example of an interrupted negative feedback loop. The method presented by Lee et al. (2012) is convenient inasmuch as existing whole-genome sequencing data can be reanalyzed to identify novel retrotransposon insertions. However, we generated similar results with per sample sequencing depth 1/12 that of Lee et al. (2012), suggesting RC-seq is more efficient for new studies specifically focused on retrotransposons.

L1-mediated insertional mutagenesis revealed *ST18* as a candidate oncogene in HCC. Numerous corroborating observations support this possibility, including (1) clonal amplification of tumor cells containing the L1 mutant *ST18*, (2) ectopic *ST18* transcription and translation in tumor not seen in adjacent nontumor liver or control liver, (3) consistent ST18 expression in transformed liver cancer cell lines, (4) frequent amplification of *ST18* in HCC nodules taken from *Mdr2*^−/−^ mice, and (5) induction of *ST18* transcription in those animals. However, we do not make any conclusion regarding the function of *ST18* as a tumor suppressor or oncogene outside of the liver and draw attention in this matter to *KLF4*, a transcriptional repressor known to function as a tumor suppressor and as an oncogene, depending on context (Rowland et al., 2005).

Overall, our results illustrate the confluence of multiple genetic aberrations in HCC, where inherited and de novo retrotransposition events form part of a wider mutational landscape. The experiments presented here and elsewhere suggest L1 activity varies substantially between individuals and cancer types (Iskow et al., 2010; Lee et al., 2012; Solyom et al., 2012). It remains to be proven whether this phenomenon correlates with prognosis, is useful in a diagnostic capacity, or can be subjected to exogenous interference in vivo. Nonetheless, we can conclude that L1-mediated retrotransposition is a potentially crucial source of mutations that can reduce the tumor suppressive capacity of somatic cells in HCC.

## Experimental Procedures

Full protocols can be found in the Extended Experimental Procedures.

### Samples

Tumor and nontumor liver tissues from 19 HCC patients with a confirmed HBV or HCV infection were provided by the Centre Hépatobiliaire, Paul-Brousse Hospital. DNA and RNA were extracted with a DNeasy Blood and Tissue Kit (QIAGEN, Hilden, Germany) and a mirVana miRNA Isolation Kit (Life Technologies, Carlsbad, CA, USA), respectively. Control liver samples from five donors were provided by the Edinburgh Sudden Death Brain and Tissue Bank. DNA and RNA were isolated through standard phenol-chloroform extraction and RNA-Bee RNA isolation reagent (Tel-Test), respectively. Samples were analyzed with approval from the French Institute of Medical Research and Health (Ref: 11-047), the East of Scotland Research Ethics Service (Ref: LR/11/ES/0022), and the Mater Health Services Human Research Ethics Committee (Ref: 1915A).

### RC-Seq Library Preparation, Sequencing, and Analysis

Multiplexed DNA sequencing libraries were constructed for HCC tumor and nontumor samples using a paired-end Illumina TruSeq Kit with substantial modifications. Briefly, 1 μg of sonicated DNA size selected for an insert size of 200–250 bp was used for each library and amplified by six cycles of ligation-mediated PCR (LM-PCR). Libraries were then pooled in groups of 4 to 6 and hybridized to an updated custom Roche NimbleGen sequence capture array comprising oligos tiling the 5′ and 3′ termini of active human retrotransposon consensus sequences (Figure 1; Table S1). Libraries were again amplified by six cycles of LM-PCR and sequenced on an Illumina HiSeq2000. After quality filtering, each read pair was assembled into a contig, aided by 2 × 150-mer sequencing and a 220 nt insert size. Read contigs were then aligned to retrotransposon consensus sequences to determine their retrotransposon donor family, aligned to the human reference genome (hg19) to determine their genomic position, and finally formed into clusters.

### PCR Validation

Germline retrotransposon insertions detected by RC-seq were first validated by a standard empty site/filled site PCR assay and then, if unsuccessful, with PCR targeting an insertion site 5′ or 3′ end. Tumor-specific insertions were characterized with a similar strategy but also incorporated 5′ and 3′ end capillary sequencing. All validation was performed on nonamplified DNA stored and handled separately from postamplification RC-seq products. Primers were designed using custom Python scripts and Primer3.

### qRT-PCR

Complementary DNA was synthesized from total RNA using random hexamers, except for L1 analyses, where a specific sense L1 primer was used. qRT-PCR was performed using a LightCycler 480 (Roche, Indianapolis, IN, USA), and values were normalized to TATA-binding protein (*TBP*). For primer sequences, see Table S8.

Extended Experimental Procedures **Sample Preparation**

Snap frozen HCC tissue samples were obtained from 19 patients who underwent surgery at the Centre Hépatobiliaire, Paul-Brousse Hospital (Table 1). The study was reviewed and approved by the Institutional Review Board (IRB) of the French Institute of Medical Research and Health INSERM (IRB Number 11-047). Specimens were obtained after surgical resection or from liver explants at transplantation. For each patient, we analyzed tumor and distal nontumor tissue (T/NT pairs). Ten patients presented HBV-induced cirrhoses, confirmed by initial pathology and by detection of HBV-surface antigen and HBx protein (see the Extended Experimental Procedures). HCV infection was confirmed for nine patients by enzyme-linked immunosorbent assay (Monolisa HCV Ag-Ab; BIO-RAD) according to the manufacturer’s instructions. Total DNA was isolated using the DNeasy blood & tissue kit (QIAGEN). Total RNA was extracted using the mirVana miRNA isolation kit (Life Technologies). DNA and RNA were eluted in 100μL nuclease-free water. RNA was quantified by spectrometry, with quality and integrity assessed on an Agilent 2100 Bioanalyzer.

Snap frozen control liver samples from 5 donors were provided by the Edinburgh Sudden Death Brain and Tissue Bank with ethical approval to be used as described (East of Scotland Research Ethics Service, Reference: LR/11/ES/0022). Autopsy revealed no evidence of liver disease. Tissue was shaved with a scalpel on dry ice and then resuspended in TE buffer with 2% SDS and 100μg/ml proteinase K at 65C until dissolved. For DNA extraction, RNase A was added to each sample to a final concentration of 10μg/ml and incubated at 37°C for 30 min. DNA was then extracted through phenol, phenol:chloroform:isoamyl alcohol (25:24:1) then chloroform:isoamyl alcohol (24:1). DNA was precipitated by adding 0.1 volume of 3 M NaOH and 2.5 volumes of 100% isopropanol and inverting tubes until DNA was completely precipitated. Precipitated DNA was spooled with a pipette tip, washed in 500μl 70% EtOH, minimally air-dried and resuspended in T0.1E buffer. RNA was extracted using RNA-Bee RNA isolation reagent (Tel-Test) and assessed for quality and quantity by NanoDrop. Further experiments at the Mater Medical Research Institute were performed with the approval of the Mater Health Services Human Research Ethics Committee (Reference: 1915A).

**RC-Seq Library Preparation and Sequencing**

Multiplexed paired-end libraries were constructed using an IlluminaTruSeq DNA sample preparation kit with the following modifications: Genomic DNA was sonicated in 120μl T0.1E using a Covaris S220 (Duty cycle: 10%, Intensity: 5, Cycles per burst: 200, Time: 150 s). Sonicated DNA was concentrated using 160μl Ampure XP beads and eluted in 55μl 10 mM Tris (pH 8.0) then quantified using a Quant-iT picogreen assay. 1 μg of sonicated DNA was used for the construction of each library. Following end repair, DNA was purified using 110μl Ampure XP beads. Libraries were size selected for 320-370bp fragments (insert size 200 – 250bp), then amplified by ligation mediated PCR (LM-PCR) comprised of 50μl 2x Phusion HF PCR Master Mix (New England BioLabs, Cat# F-531L), 2μM LM-PCR primers (IDT) (LM-PCR 1.0: 5′-AATGATACGGCGACCACCGAGA-3′, LM-PCR 2.0:5′-CAAGCAGAAGACGGCATACGAG-3′), 30μl Library and PCR grade water to 100μl. Amplification proceeded via the following program: 98°C for 30 s, 6 cycles of 98°C for 10 s, 60°C for 30 s and 72°C for 30 s, final extension at 72°C for 5 min and hold at 10°C. Amplified libraries were purified using 110μl Ampure XP beads, and eluted in 30μl molecular grade water. Libraries were quantified, and insert size confirmed using an Agilent Bioanalyzer 2100 with a DNA1000 chip (Cat# 5067-1504).

For sequence capture, equimolar quantities of 4 to 6 libraries were pooled, with T/NT pairs kept together, to give a total quantity of 800ng DNA. Sequence capture was performed using a Roche NimbleGen SeqCap EZ Library SR kit with Plant Capture Enhancer (PCE) in place of COT DNA, and custom blocking oligos (IDT) (TS-Uni: 5′-AAT GAT ACG GCG ACC ACC GAG ATC TAC ACT CTT TCC CTA CAC GAC GCT CTT CCG ATC^∗^/3InvdT/-3′ and TS-HexI: 5′-CAA GCA GAA GAC GGC ATA CGA GAT III III GTG ACT GGA GTT CAG ACG TGT GCT CTT CCG ATC T ^∗^/3InvdT/-3′) in place of TS-HE Universal and TS-INV-HE Index oligos. Libraries were again amplified by LM-PCR with the same conditions as above except with 200μl volumes and primers at 1μM final concentration. Library capture was confirmed with an Agilent DNA1000 chip. Libraries were sequenced in multiplex using six lanes of an Illumina HiSeq2000 (ARK-Genomics, Edinburgh and University of Queensland Centre for Clinical Genomics, Brisbane).

**RC-Seq Computational Analysis**

2x150-mer sequencing of ∼220nt inserts enabled assembly of each read pair into a “contig” before genomic alignment. This substantially improved the existing computational interrogation of RC-seq reads (Baillie et al., 2011). Briefly, reads were trimmed from their 5′ and 3′ ends to remove any bases with quality <10, then assembled into contigs using FLASH (Magoč and Salzberg, 2011) with default parameters. Read contigs were then aligned to Illumina linker sequences by BLAST to remove possible ligation artifacts (-m 8 -a 8 -F F, minimum score 22) and then aligned to retrotransposon consensus sequences (Baillie et al., 2011) with LAST (Kiełbasa et al., 2011) using the parameters –s2 –l10 –d30 –q3 –e30. Reads were retained if a suitable match was found (identity ≥95%, ≥33nt alignment spanning one contig end) and annotated as performed previously (Baillie et al., 2011). At this stage, read contigs were arranged with a 5′ nonretrotransposon section (≥33nt) followed by a 3′ retrotransposon section (≥33nt), as illustrated in Figure 1.

Rigorous genomic alignment was then performed in sequential fashion against hg19, starting with SOAP2 (Li et al., 2009) (parameters -M 4 -v 2 -r 1). Reads that could be aligned in full in this step detected reference genome insertions. Reads that could not be aligned were mapped again to hg19 using LAST (s2 –l11 –d30 –q3 –e30), which excels in reporting split alignments found for translocations and, for RC-seq, where one end of the assembled read contig maps to one location on the genome and the other end maps elsewhere. Any read with an alignment of the nonretrotransposon section plus 10nt was removed as a potential molecular chimera. The remaining reads with a uniquely mapped nonretrotransposon section were used to indicate the nucleotide position and strand of nonreference genome insertions. These were then formed into clusters as described previously (Baillie et al., 2011) except opposing clusters were only joined if separated by ≤100nt. This strategy resulted in single nucleotide resolution of novel insertion breakpoints and a more than 10-fold reduction in CPU time per library. A complete list of annotated novel insertions supported by at least two unique amplicons separated by ≥5nt (the minimum threshold for reporting) is provided in Table S3.

**PCR Validation of Novel Insertions**

Germline retrotransposon insertions detected by RC-seq were assayed by PCR using a standard empty site / filled site assay. Primers were positioned on either side of the insertion site. For examples with a predicted filled site > 1kb in length, or shorter insertions where a filled site was not detected using the standard assay, additional retrotransposon specific primers were designed and paired with the existing insertion site primers. PCR reactions contained 2U MyTaq hot-start DNA polymerase (Bioline #BIO-21112), 1X PCR buffer, 1μM of each primer and 10ng genomic DNA in a 25μL reaction. The following cycling conditions were used: 95°C for 2 min, then 35 cycles of 95°C for 15 s, 60°C for 15 s, 72°C for 1 min, followed by a single extension step at 72°C for 10 min. Optimization in some cases required adjusted annealing temperatures, cycle number, or changing polymerase enzymes. If multiple PCR products similar to the correct size were observed capillary sequencing was used to clarify validation.

Tumor-specific insertions detected by RC-seq were assessed using a strategy similar to that used for germline insertions except, in this case, additional primers were generated to characterize insertion 5′ and 3′ ends (Table S6). All products were capillary sequenced using an ABI3730 (GenePool, Edinburgh and AGRF, Brisbane). All primers were designed using custom Python scripts and Primer3 (Rozen and Skaletsky, 2000). Input DNA for all PCR validation reactions, for both germline and tumor-specific insertions, was stored and handled separately to postamplification Illumina libraries.

**DNA Methylation Analyses**

We followed previously described protocols to analyze the level of L1 promoter methylation (Coufal et al., 2009; Wissing et al., 2012). Briefly, 2μg of genomic DNA was bisulfite converted using an Epitect Kit (QIAGEN) following manufacturer instructions. After purification, 300-500ng of bisulphite converted genomic DNA was used as template in a PCR reaction with 10Us Taq polymerase (Roche Expand High Fidelity Taq), 0.2 mM dNTPs (Invitrogen), and 200ng of L1_Bis-F and L1_Bis-R primers (see Table S8) in a 50ul reaction. The following cycling conditions were used: 2 min at 95°C, then 35 cycles of 30 s at 94°C, 30 s at 54°C, 60 s at 72°C, followed by a single extension step at 72°C for 5 min. Negative controls were included at each step using RNA/DNA-free water (Invitrogen). PCR fractions were then resolved on agarose gels, the ∼350bp amplification band excised, purified using a QIAquick gel extraction kit (QIAGEN), and cloned into the pGEM-T Easy Vector (Promega). More than 20 independent clones per sample were capillary sequenced using universal primers. Sequences were aligned to a mock bisulphite converted consensus L1-Ta sequence (L1.3, accession L19088.1) using ClustalX (Thompson et al., 1997) and the methylation status of CpG dinucleotides was scored by hand. The 7 sequences with the highest identity compared to the consensus sequence were used to graphically represent the overall level of L1 promoter methylation, as shown in Figure S4C. Chi-square tests were used to calculate the significance of the proportion of methylated and unmethylated CpG dinucleotides in each sample or group.

**Cell Culture**

Human hepatocellular cell lines Huh7, HepG2, PLC/PRF/5 and HeP3B were a kind gift from Dr. Bakary Sylla (International Agency for Research on Cancer, Lyon, France). Cells were cultured in DMEM-F12 (1:1) media supplemented with 10% fetal bovine serum, 2 mM Glutamax, 0.5 mM sodium pyruvate and 1% nonessential amino acids at 37°C and 5% CO_2_.

**Immunoblot**

Tissues or cell line pellets were lysed in western lysis buffer containing 50 mM HEPES pH 7.1, 1% Triton X-100, 50 mM NaCl, protease inhibitor cocktail (Roche #11836 153001) and phosphatase inhibitors cocktails (Sigma #P2850, #P5726). Protein was estimated by Bradford method and 30μg of protein extracts were loaded on 7.5% sodium dodecyl sulfate-polyacrylamide gel (SDS-PAGE). After electrophoresis, proteins were transferred to polyvinylidene difluoride membranes (Millipore #IPVH00010). Membranes were blocked with 5% milk and then immunoblotting was done with the required primary antibody (anti-ST18 (Abcam #ab127900, 1:1000), anti-MCC (Santa Cruz #sc-135982,1:500), anti-CTNNB1 (Santa Cruz #sc-7199, 1:1000), anti-GAPDH (Abcam #ab125247,1:5000)) followed by peroxidase-conjugated secondary anti-rabbit (Cell Signaling #7074, 1:5000) or anti-mouse (Cell Signaling #7076, 1:5000) antibody and visualized using an enhanced chemiluminescence detection system (GE Amersham #RPN2132).

**Immunohistochemistry**

4 μm-thick sections of formalin-fixed, paraffin-embedded liver samples were de-waxed in xylene prior to rehydration. Antigen retrieval was performed by boiling slides in 1 mM EDTA pH 8.0 for 20 min (for CTNNB1 staining) and in 10 mM citrate buffer pH 6.0 for 10 min (for ST-18 staining). Sections were incubated for 1 hr at room temperature with mouse CTNNB1 monoclonal antibody (1:300 dilution, clone 17C2, Abcys) or rabbit ST-18 polyclonal antibody (1:200 dilution, Abcam Ab86563). Incubation with primary antibody was followed by incubation with either peroxidase-conjugated donkey anti-mouse or rabbit antibody. Immunoreactive staining was detected using the Dako Envision System HRP (DAKO, CA, USA). Nuclei were counterstained with hematoxylin.

**Chromatin Immunoprecipitation**

Huh7 cells were fixed with 1% formaldehyde for 10 min at room temperature, fixing was neutralized by 125 mM glycine and cells were harvested using a cell scraper. 10^7^ cells were used per group and were sonicated in a Covaris S220 at 4°C for 20 min. 10 μg anti-ST18 antibodies (Abcam #ab86563 and #ab127900) were utilized for immunoprecipitation, rabbit IgG (Millipore #12-370) was used as a control. Coimmunoprecipitated chromatin fragments were reverse crosslinked and analyzed for target and nontarget regions using quantitative real-time PCR. Primers are given in Table S8.

**Expression Analysis**

Total RNA was treated with a Turbo DNA-free kit (Ambion #AM1906) and reverse transcribed with the SuperscriptIII first-strand synthesis system (Invitrogen #18080-044). For L1 analyses, cDNA synthesis required a specific sense L1 primer. For all other analyses, cDNA was prepared using random hexamers. qRT–PCR was performed using LightCycler 480 SYBR green mix (Roche #04707516001) or LightCycler probe master mix (Roche #04707494001) according to the manufacturer’s instructions (primer sequences are given in Table S8) and run on a LightCycler 480 (Roche). *TBP*, *GAPDH* and *HPRT* were assessed as normalization controls. *TBP* provided the most precise measurements across individuals and was selected as the control for all qRT-PCR experiments presented here.

**HBV Detection**

PCR reactions contained 0.5μL MyTaq DNA polymerase (Bioline), 1X PCR buffer, 1μM of each primer and 100ng genomic DNA in a 50μL reaction volume. The following cycling conditions were used: 94°C for 2 min, then 35 cycles of 94°C for 15 s, 55°C for 15 s, 70°C for 10 s, followed by a single extension step at 70°C for 10 min. Primer sequences are given in Table S8.

**ST18 Copy Number and Expression Analysis in Mdr2^−/−^ Mice**

The *Mdr2*^−/−^ mouse is an established animal model of inflammation driven HCC (Mauad et al., 1994). A total of 27 nodules representing different time points of tumor progression (Table S7) were collected from 10 *Mdr2*^−/−^ mice (7 males + 3 females) sacrificed at 13-16 months, together with matched normal tissue (kidney). Stage of disease and tumor content were assessed through pathological inspection. 4 nodules were found to not include HCC cells. Genomic DNA was extracted from samples using DNeasy Tissue kit (QIAGEN) according to the manufacturer’s protocols. Total DNA concentration and quantity were assessed by measuring absorbance at 260nm with a NanoDrop 1000 Spectrophotometer (Thermo Fisher Scientific). *ST18* copy number was assessed by quantitative real-time PCR using a TaqMan copy number assay (gene probe: Mm00040629_cn) on a 7900HT Fast Real-Time PCR System (Applied Biosystems) with sequence detection systems software 2.2.2. *TERT* (Applied Biosystems, part number 4458373) was used as a reference. All samples were plated in quadruplicates with 20ng DNA for each reaction. CNV calling was done with CopyCaller v2.0 (Applied Biosystems) and normalized to kidney. For qRT-PCR, total RNA was extracted from nodules and wild-type mouse liver samples using an RNeasy kit (QIAGEN) according to the manufacturer’s instructions. 500ng total RNA from each sample was then used for cDNA synthesis with ImProm-II Reverse Transcriptase (Promega). 1 μl cDNA from each reaction was used for qRT-PCR using the mouse ST18 primers listed in Table S8. qRT-PCR (SYBR-green) analysis was performed on an Applied Biosystems 7500 Real-time PCR system. Values were normalized to *TBP*.

## Accession Numbers

RC-seq FASTQ files were deposited in the European Nucleotide Archive (ERP001476).

## Supplemental Information

Table S1. RC-Seq Sequence Capture Probes, Related to Figure 1 “Position” refers to the first nucleotide position (base 0) in the corresponding retrotransposon consensus sequence.

Table S2. RC-Seq Filtering and Mapping Statistics, Related to Table 1 For further analysis pipeline information, see the Extended Experimental Procedures.

Table S3. Complete List of Nonreference Genome Insertions Detected by RC-Seq, Related to Table 1 “Sense start” and “Sense stop” refer to the start and stop positions of an RC-seq read cluster arranged in sense to the genome. “Antisense start” and “Antisense stop” refer to a cluster arranged antisense to the genome. Examples with sense and antisense RC-seq clusters depict simultaneous 5′/3′ detection of an insertion. “Pooled blood” lists whether the insertion was detected in previously published pooled blood RC-seq (Baillie et al., 2011). “Donor sense” records the retrotransposon consensus start position (base 0) of sense insertions (negative values or 0) or the stop position of antisense insertions (positive values). “Donor antisense” records the retrotransposon consensus stop position of sense insertions (positive values) or the start position of antisense insertions (negative values or 0). “Private” is yes (Y) if an insertion is only found in one donor and not in pooled blood or an existing database. “Annotation” records the tissue in which an insertion was detected (T/NT: tumor and nontumor liver, T: tumor only, NT: nontumor only). For instance, a tumor-specific insertion would be annotated as Y for “Private” and T for “Annotation.”

Table S4. List of Full-Length, Nonreference Genome L1 Insertions Not Previously Annotated, Related to Figure 2 Column labels are as for Table S3.

Table S5. Nonreference Genome Germline Insertions Validated by Site-Specific PCR, Related to Figures 2 and 3 Different tabs are included for a set of 200 random insertions and for a selection of 36 insertions in cancer candidate genes. “RC-seq information” is a condensed version of the same data presented in Table S3 for each insertion. “Validation summary” lists primers and validation results.

Table S6. List of Somatic Insertions Validated by Site-Specific PCR and Capillary Sequencing, Related to Figure 6 “RC-seq information” is as for Table S4. Insertions validated as germ line or not validated are listed in the bottom half of the table. All validated insertions were tumor specific except for the liver-specific example highlighted in Figure S3. “Validation summary” contains information on whether the mutant allele (retrotransposon) and null allele were detected by PCR, the validation result, the sequences of insertion site primers (ISPs) and other primers (OPs), and the combination of these primers producing detectable amplification. “Characterization” includes the capillary sequences of confirmed somatic insertions, TSD sizes, the L1 endonuclease recognition motif, the start position of truncated insertions, and notes regarding the features observed at the insertion site.

Document S1. Tables S7 and S8

Document S2. Article plus Supplemental Information
