# Supplementary material for: Endogenous Retrotransposition Activates Oncogenic Pathways in Hepatocellular Carcinoma
Source: Cell. 2013 Mar 28;153(1):101–11. doi: 10.1016/j.cell.2013.02.032 (PMC3898742; doi:10.1016/j.cell.2013.02.032)
Supplement: Document S1. Tables S7 and S8 [file mmc7.pdf]

Table S7. *Mdr2*$^{-/-}$ Mouse TaqMan qPCR *ST18* Copy Number Analysis, Related to Figure 6

**Mouse ID**	**Nodule**	**Sex**	**Age (months)**	**Nodule size (cm)**	**Histology (% HCC)**	***ST18* copy number**
215	1	M	14	1.8	80	2
51505	1	M	14	0.7	0	2
51505	2	M	14	-	80	2
60123	1	M	14	0.6	0	2
218	1	M	15	1	50	3
218	2	M	15	3	90	2
218	3	M	15	1	70	2
58853	1	M	15	1.5	10	2
58853	2	M	15	4	10	2
58853	3	M	15	1.7	60	2
58853	4	M	15	1	10	2
58163	1	M	15	4	10	2
58163	2	M	15	1.3	10	2
58163	3	M	15	3	70	4
58163	4	M	15	3	70	2
51509	1	M	16	1.5	20	2
51509	2	M	16	1.1	20	2
51509	3	M	16	0.8	20	2
51509	4	M	16	1.1	0	2
60400	1	F	13	0.9	60	2
60400	2	F	13	1.4	40	2
60400	3	F	13	0.5	10	2
60400	4	F	13	0.5	10	2
52686	1	F	15	1	20	2
52686	2	F	15	0.7	50	3
52687	1	F	15	1.3	0	2
52687	2	F	15	0.9	20	3

27 nodules from 10 animals were assessed for HCC stage by pathological examination and *ST18* CNV was detected by TaqMan qRT-PCR (see Extended Experimental Procedures). HCC was found in 23 nodules. 4 HCC nodules from 4 different animals presented amplified *ST18* (highlighted in green). 3 out of 4 nodules (75%) with amplified *ST18* showed high % HCC (≥50%). *ST18* copy number was 2 for all remaining nodules.

Table S8. Oligonucleotides for L1 Promoter Bisulphite Sequencing, HBV Detection, qRT-PCR, and ChIP-qPCR, Related to Experimental Procedures

Oligonucleotide name	Oligonucleotide sequence
**L1 promoter bisulphite sequencing**	
L1_Bis-F	AAGGGGTTAGGGAGTTTTTTT
L1_Bis-R	TATCTATACCCTACCCCCAAAA
**HBV detection**	
HBV_s_459-F	AGGTATGTTGCCCGTTTGTCCT
HBV_s_1001-R	CAGCAAAGCCCAAAAGACCCAC
HBV_x_1174-F	TGCCAAGTGTTTGCTGACGC
HBV_x_1627-R	GTTCACGGTGGTCTCCATG
**Human genes qRT-PCR**	
TBP-F	GCAAGGGTTTCTGGTTTGCC
TBP-R	GGGTCAGTCCAGTGCCATAA
MCC-F	TATGGAAACGACTCCTCGGC
MCC-R	TCTCATGAGGTGGGACTGCT
ST18-F	CGTGCCAGCTCTTATAGCTACG
ST18-R	TATTTCGGCTCCCTTGGCAT
EFHD1-F	GCTCATTTTCCACAAGGCCG
EFHD1-R	TGACAAGGCTTGGACCTTGG
SLC2A1-F	GGCTTCTCCAACTGGACCTC
SLC2A1-R	CCGGAAGCGATCTCATCGAA
PHGDH-F	CCAATGGGAACAGCCTCAGT
PHGDH-R	CTCCCATTTGCCGTCCTTCA
**Human L1 TaqMan qRT-PCR**	
L1-5'-UTR-F	ACAGCTTTGAAGAGAGCAGTGGTT
L1-5'-UTR-R	AGTCTGCCCGTTCTCAGATCT
L1-5'-UTR-probe	VIC-TCCCAGCACGCAGC-MGB
L1-ORF2-F	TGCGGAGAAATAGGAACACTTTT
L1-ORF2-R	TGAGGAATCGCCACACTGACT
L1-ORF2-probe	VIC-CTGTAAACTAGTTCAACC-MGB
L1-RT	ACATGTGCACATTGTGCAGGTTAG
**Mouse *ST18* qRT-PCR**	
mouse_ST18-F	TGGATGCCGAGGTTGAAGAT
mouse_ST18-R	TCAGCTCTCCTCTTCTTCGC
mouse_TBP-F	CTGGAATTGTACCGCAGCTT
mouse_TBP-R	ATGATGACTGCAGCAAATCG
**Human ST18 ChIP-qPCR**	
ChIP-ST18-F	GCACACATCCCCAAAAGAAT
ChIP-ST18-R	CCTGCTATGAATTTTCCATGA
ChIP-GAPDH-F	TCGACAGTCAGCCGCATCT
ChIP-GAPDH-R	CTAGCCTCCCGGGTTTCTCT
